# Modeling the Combined Effects of Straw Returning, Urease Inhibitors, and Nitrogen Split Application on Rice Yield and Ammonia Volatilization in Purple Soil Area

**DOI:** 10.3390/plants14121744

**Published:** 2025-06-06

**Authors:** Tianxiang Xu, Hong Wang, Huirong Hao, Chaowen Lin, Kelin Hu

**Affiliations:** 1 College of Land Science and Technology, China Agricultural University, Key Laboratory of Arable Land Conservation (North China), Ministry of Agriculture and Rural Affairs, Beijing 100193, China; 2 Institute of Agricultural Resources and Environment, Sichuan Academy of Agricultural Sciences, Chengdu 610066, China

**Keywords:** straw returning, urease inhibitors, N split application ratio, rice yield, NH_3_ volatilization, WHCNS model

## Abstract

The application of urease inhibitors (UIs) and optimizing nitrogen (N) split application ratio (NSR) can both minimize ammonia (NH_3_) volatilization and increase rice yield. However, few studies have analyzed the combined effects of these two practices with straw returning on rice yield and NH_3_ volatilization. In this study, based on a field experiment involving rice yield, aboveground dry matter (ADM), crop N uptake (N_upt_), and NH_3_ volatilization from 2018 to 2019 in Sichuan Basin, China, the WHCNS (soil water heat carbon nitrogen simulator) model was used to simulate the effects of straw returning, UI, and NSR on rice growth and NH_3_ volatilization. The results showed that the WHCNS model performed well in simulating rice growth and NH_3_ volatilization. With straw return amount exceeding 4 t ha^−1^, rice yield increased slowly or stabilized, while N_upt_ and NH_3_ volatilization continued to increase. Increasing the panicle fertilizer (PF) proportion enhanced N_upt_ during the PF stage, thereby promoting yield improvement. The NSR_3_ (a 1:1:3 ratio of base fertilizer, tiller fertilizer, and PF) achieved the highest yield, exceeding that of 2:1:2 by 0.29, 0.23, and 0.08 t ha^−1^ at straw return amounts of 2, 3, and 4 t ha^−1^, respectively. However, the effects of UI on N_upt_ and yield enhancement were limited. Furthermore, optimized NSR and the application of UI reduced NH_3_ volatilization during the basal or tiller fertilizer stages, leading to an average decrease of 5.5% and 8.5% in total NH_3_ volatilization, respectively. Meanwhile, the increase in straw return amount reduced the NH_3_ volatilization reduction effects of both practices. Overall, the combination of NSR_3_ and UI with the straw return amount of 3 t ha^−1^ was the optimal practice for balancing food security and environmental benefits in purple soil area.

## 1. Introduction

As a major staple crop, rice (*Oryza sativa* L.) serves as a crucial dietary component, supplying essential calories to more than 50% of the global population [1,2]. Rice consumption is projected to reach 590 million tons by 2040, driven by increasing global population demands [3]. Meanwhile, nitrogen (N) inputs are expected to increase to satisfy crop N requirements [4]. However, the corresponding N loss is projected to have substantial global impacts, potentially costing up to 3% of the world’s gross domestic product [5]. The primary form of N loss in paddy field is ammonia (NH_3_) volatilization, which can account for up to 50% of the applied N fertilizer [6]. NH_3_ volatilization not only contributes to atmospheric haze formation but also deposits into water bodies and terrestrial ecosystems, leading to eutrophication of water bodies, and soil acidification, ultimately posing significant risks to ecosystems and human health [6,7]. Consequently, it is imperative to optimize field management practices to ensure food security and minimize NH_3_ volatilization.

Straw returning, the application of urease inhibitors (UIs), and optimizing the N split application ratio (NSR) are important field management practices for increasing rice yield and minimizing NH_3_ volatilization [8,9,10]. Previous research has widely demonstrated that straw returning can enhance crop N uptake (N_upt_) and increase yield [9,11]. Straw returning not only elevates soil nutrient content but also enhances soil nutrient retention capacity through increased soil organic carbon, thus enhancing the development of rice grains and panicles, subsequently increasing rice yield [12,13]. Meanwhile, straw returning contributes to improving the poor soil structure in paddy field, thus promoting root growth and increasing water absorption by plants [14,15]. However, straw returning leads to a competition between microorganisms and plants for N absorption, and excessive straw return amount may increase the risk of soil-borne diseases, thereby threatening crop growth [16]. The concentration of NH_4_^+^-N and pH in floodwater are the most critical factors affecting NH_3_ volatilization in paddy fields [17]. On the one hand, straw returning increases soil organic acid content, resulting in a decreased pH. It also boosts microbial activity, promoting the conversion of inorganic N to organic N, which collectively suppresses NH_3_ volatilization [18]. On the other hand, straw returning increases the NH_4_^+^-N concentration in floodwater, facilitating the NH_3_ volatilization process [10,17]. Given these opposing effects of straw returning on rice yield and NH_3_ volatilization, identifying an optimal straw return amount is essential. Additionally, the influence of straw returning on NH_3_ volatilization and N_upt_ during various rice growth stages remains unclear.

Urea is the predominant N fertilizer in worldwide agricultural systems. However, it undergoes rapid hydrolysis after application, leading to substantial N loss through NH_3_ volatilization and ultimately resulting in low N use efficiency (NUE) [5,7]. UIs suppress soil urease activity, slowing the hydrolysis of urea and thus serving as an effective measure to reduce NH_3_ volatilization [19]. A global study on UIs showed that it reduced NH_3_ volatilization by 51% worldwide, with even more pronounced reductions in regions with higher NH_3_ emissions [20]. In addition, UIs can reduce the indirect nitrous oxide emissions caused by NH_3_ deposition [21]. The UIs can regulate the transformation of various N forms in the soil, enhancing soil N supply capacity to better meet the N demands of crop during each growth stage [10,19]. Similarly, optimizing the NSR is also a widely used strategy to optimize the allocation of N fertilizer supply, thus increasing yield and reducing NH_3_ volatilization [8,22]. In China, farmers frequently apply substantial N fertilizer during the basal fertilizer (BF) stage, which misaligns with the crop’s N requirements across different growth stages [23]. During early growth, limited root development reduces the crop’s capacity to absorb N, while insufficient N supply in later stages restricts grain and panicle development, ultimately lowering yield [12,23]. Increasing the proportion of N fertilizer in later growth stages can more effectively aligns with the crop’s N demand, while also reducing N loss, especially by limiting NH_3_ volatilization during the BF stage [8,22]. However, research analyzing the combined effects of straw returning, UIs, and NSR on rice yield and NH_3_ volatilization in paddy fields remains relatively limited.

Traditional field experiments have limitations in monitoring crop growth and N loss throughout the entire growing season, and also require significant time and cost investments. Process-based models, which employ a series of mathematical equations to quantify crop growth, soil water dynamics, and N transformation, offer an effective solution to these limitations. For example, models such as DNDC [24], Hydrus [25], Oryza [26], and CERES-Rice [27] have been widely applied to evaluate rice productivity and N loss across diverse cultivation practices. Liang et al. [28] developed the Soil Water Heat Carbon Nitrogen Simulator (WHCNS), a process-based model based on field management practices in China, which has been successfully applied to simulate rice development and N loss in paddy fields [8,29]. However, the model has not yet simulated the combined effects of straw returning, UI, and NSR on rice yield and NH_3_ volatilization in paddy fields.

In this study, we employed the WHCNS model to conduct a comprehensive analysis of the impacts of straw return amount, UI, and NSR on rice yield, N_upt_ and NH_3_ volatilization based on two years of field plot experiments. The objectives of this study are (1) to reveal the combined effects of straw returning, UI, and NSR on rice yield and NH_3_ volatilization, and (2) to determine the optimal field management measures in the study area. This study will provide a theoretical foundation for the efficient utilization of straw, as well as for balancing food security and environmental benefits.

## 2. Results

### 2.1. Model Calibration and Evaluation

The WHCNS model demonstrated good performance in simulating rice yield, aboveground dry matter (ADM), and N_upt_ (Table 1; Figures S1–S3). The *nRMSE* of rice yield was less than 10% in both years. Meanwhile, the *IA* and *NSE* of rice yield were larger than 0.90 and 0.60 in both years, respectively. The simulated and observed rice yield increased rapidly with the increase in straw return amount when the straw return amount was less than 5 t ha^−1^. However, when the straw return amount exceeds 5 t ha^−1^, the yield increased slowly or remained relatively stable. Among different straw returning treatments, only significant difference was observed in rice yield between US_2_ and US_8_ in 2019 (*p* < 0.05). Additionally, the contribution of UI to yield improvement was limited, and no significant differences were observed in rice yield between treatments with and without UI application under the same straw return amount (Figure S1). The *nRMSE* of ADM were less than 10% in both years. Furthermore, the *IA* and *NSE* of ADM remained above 0.90 and 0.50 in both years, respectively. For N_upt_, the *nRMSE* was less than 15% with *IA* and *NSE* exceeding 0.80 and 0.20 in both years, respectively. The simulated and observed ADM and N_upt_ both showed an increasing trend with the increase in straw return amount. The ADM of CK was significantly lower than that of N input treatments (*p* < 0.05), while there were no significant differences between different N input treatments. The N_upt_ of US_8_ and UIS_8_ were significantly higher than that of US_2_, US_5_, UIS_2_, and UIS_5_ in 2019, while only significant difference was observed between UIS_8_ and UIS_2_ in 2018 (*p* < 0.05). Similarly, the effect of UI on ADM and N_upt_ was also limited, and no significant differences were observed in ADM and N_upt_ between treatments with and without UI application under the same straw return amount (Figures S2 and S3).

The WHCNS simulations of NH_3_ volatilization also exhibited strong agreement with the observed data (Table 1; Figures S4–S6). The WHCNS model effectively simulated the dynamic variation in NH_3_ volatilization, particularly capturing the peak values following each fertilization event. The *nRMSE* in 2018 and 2019 was 7.2% and 15.1%, respectively. In addition, the *IA* and *NSE* exceeded 0.90 and 0.70, respectively. The simulated and observed NH_3_ volatilization showed an increasing trend with the increase in straw return amount, and the application of UI reduced NH_3_ volatilization. However, there were no significant differences in cumulative NH_3_ volatilization between different N input treatments (Figure S6).

### 2.2. Rice Yield and Crop N Uptake

**Figure 1:**
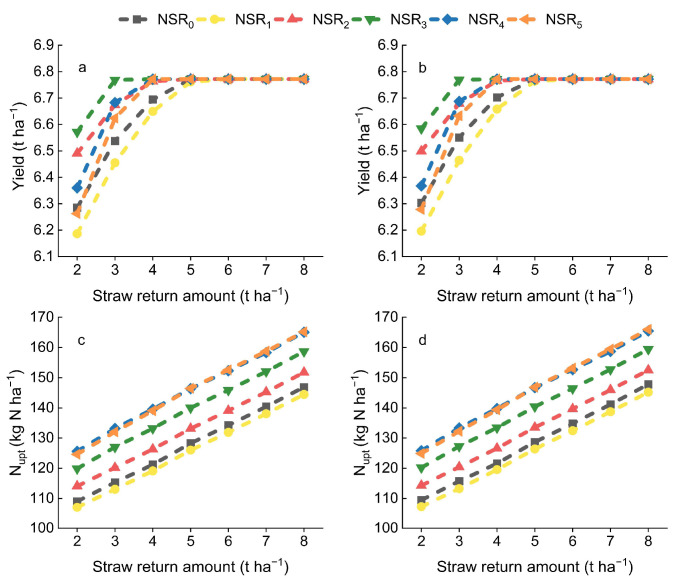
Rice yield and crop N uptake (N_upt_) throughout the growing season. NSR_0_, NSR_1_, NSR_2_, NSR_3_, NSR_4_, and NSR_5_: N split application ratios of 2:1:2, 1:2:2, 2:3:5, 1:1:3, 1:2:7, and 2:1:7, respectively; (**a**,**c**): without UI application; (**b**,**d**): with UI application.

Increasing the PF proportion or straw return amount within a specific range could both increase rice yield (Figure 1). However, the application of UI exhibited negligible increase in rice yield (Figure 1). Among all NSR, NSR_3_ demonstrated the highest rice yield, and the maximum rice yield could be achieved at a straw return amount of 3 t ha^−1^ with NSR_3_. Specifically, the rice yield of NSR_3_ exceeded that of NSR_0_ by 0.29, 0.23, and 0.08 t ha^−1^ at straw return amounts of 2, 3, and 4 t ha^−1^, respectively. Rice yield reached their maximum at a straw return amount of 4 t ha^−1^ with NSR_2_, NSR_4_, and NSR_5_, while the maximum rice yield was attained at a straw return amount of 5 t ha^−1^ with NSR_0_ and NSR_1_.

**Figure 2:**
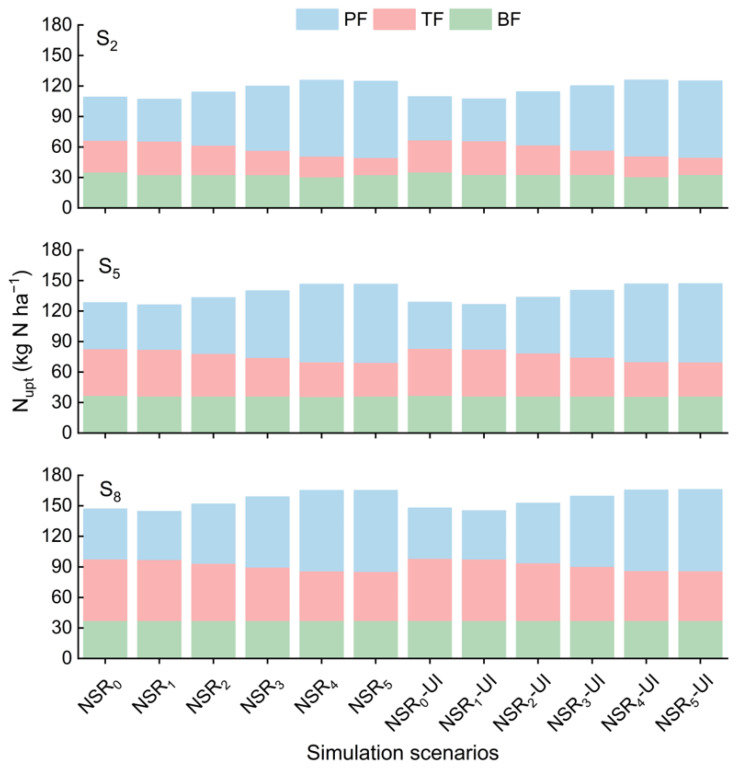
Crop N uptake (N_upt_) during different stages at straw return amounts of 2 t ha^−1^ (S_2_), 5 t ha^−1^ (S_5_), and 8 t ha^−1^ (S_8_). BF: basal fertilizer, TF: tiller fertilizer, PF: panicle fertilizer. NSR_0_, NSR_1_, NSR_2_, NSR_3_, NSR_4_, and NSR_5_: N split application ratios of 2:1:2, 1:2:2, 2:3:5, 1:1:3, 1:2:7, and 2:1:7, respectively; UI: urease inhibitor.

Similarly, N_upt_ increased with the increasing PF proportion, while the application of UI exhibited negligible increase in N_upt_. Furthermore, N_upt_ exhibited a consistent upward trend with the increased straw return amount (Figure 1). As the PF proportion increased, N_upt_ during the PF stage consistently augmented, while that during the TF stage diminished (Figure 2). However, a decreased BF proportion only resulted in a decrease in N_upt_ during the BF stage at straw return amounts of 2 and 5 t ha^−1^; at a straw return amount of 8 t ha^−1^, N_upt_ during the BF stage remained unchanged. The enhancement of increasing the PF proportion on N_upt_ during the PF stage also gradually weakened with the straw return amount increased. Furthermore, an increase in straw return amount increased N_upt_ across all stages, with the most pronounced effect observed in the TF stage.

### 2.3. NH_3_ Volatilization

**Figure 3:**
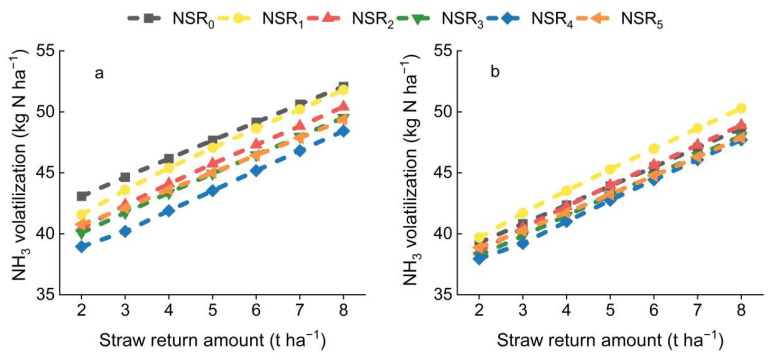
NH_3_ volatilization throughout the growing season. (**a**): without UI application; (**b**): with UI application. Symbol explanation can be found in Figure 2.

As illustrated in Figure 3, increasing the PF proportion and the application of UI both contributed to mitigating NH_3_ volatilization, while straw returning led to a continuous increase in NH_3_ volatilization. Specifically, under the conditions of NSR_0_, NSR_1_, NSR_2_, NSR_3_, NSR_4_, and NSR_5_, the application of UI reduced NH_3_ volatilization by 8.5%, 3.9%, 4.0%, 4.1%, 2.0%, and 4.1%, respectively. Additionally, NSR_1_, NSR_2_, NSR_3_, NSR_4_, and NSR_5_ exhibited decreases in NH_3_ volatilization of 1.6%, 4.5%, 6.2%, 9.4%, and 5.8% compared to NSR_0_ in the absence of UI application, respectively. However, NSR_2_, NSR_3_, NSR_4_, and NSR_5_ exhibited decreases in NH_3_ volatilization of 0.2%, 1.9%, 2.9%, and 1.5% compared to NSR_0_ under the conditions of UI application, respectively. Conversely, NSR_1_ exhibited an increase in NH_3_ volatilization by 2.7% compared to NSR_0_ under the conditions of UI application. Notably, the effectiveness of increasing PF proportion and the application of UI in reducing NH_3_ volatilization became more pronounced as the straw return amount decreased.

**Figure 4:**
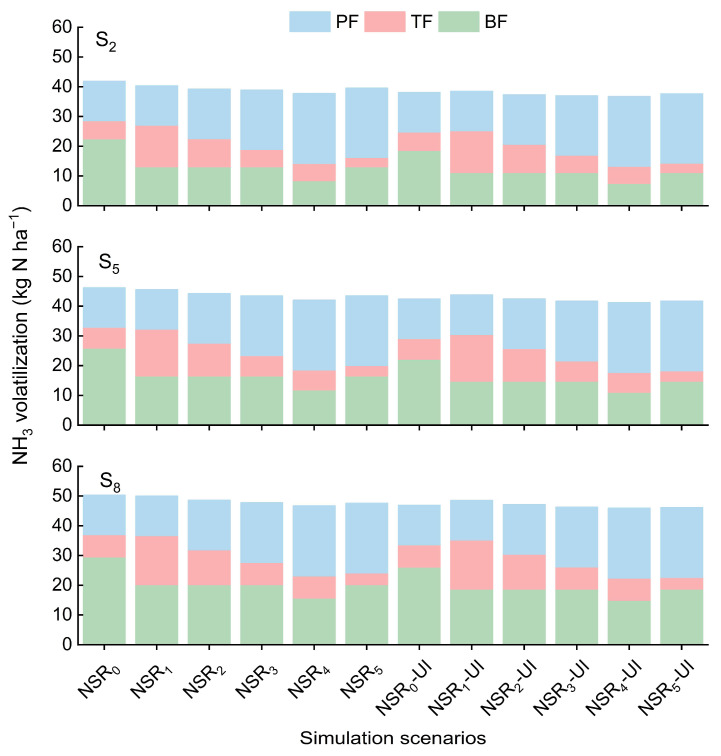
NH_3_ volatilization during different stages at straw return amounts of 2 t ha^−1^ (S_2_), 5 t ha^−1^ (S_5_), and 8 t ha^−1^ (S_8_). Symbol explanation can be found in Figure 2.

Although increasing the PF proportion increased NH_3_ volatilization during the PF stage, it decreased NH_3_ volatilization during the BF or TF stages, ultimately leading to a reduction in NH_3_ volatilization throughout the entire growth period (Figure 4). Additionally, the impact of UI and straw returning on NH_3_ volatilization was primarily observed during the BF stage, with limited effects during the TF and PF stages.

### 2.4. N Use Efficiency

**Figure 5:**
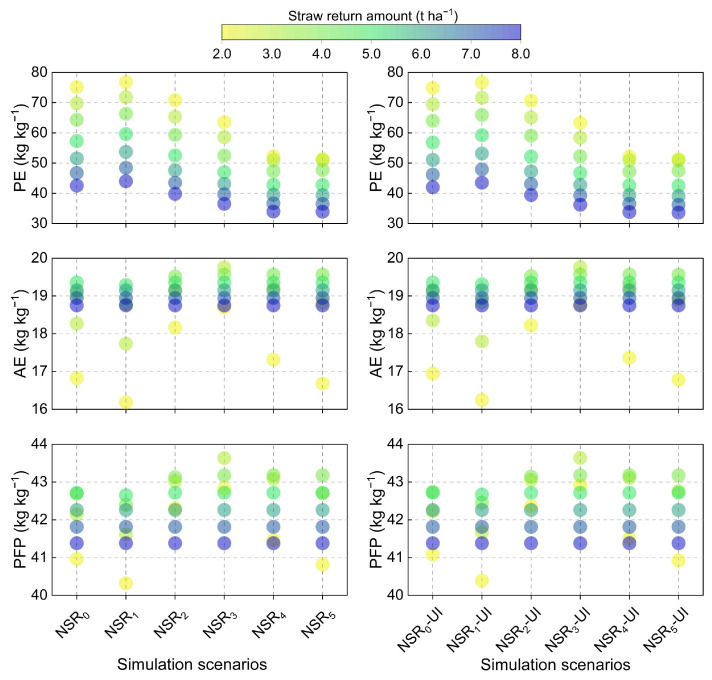
N physiological efficiency (PE), agronomic efficiency (AE), and partial factor productivity (PFP) under different simulation scenarios. Symbol explanation can be found in Figure 2.

The application of UI failed to elicit an augmentation in rice yield and N_upt_, consequently resulting in no discernible improvement in N physiological efficiency (PE), agronomic efficiency (AE), and partial factor productivity (PFP) (Figure 5). The PE of NSR_0_, NSR_1_, NSR_2_, NSR_3_, NSR_4_, and NSR_5_ was 58.0, 59.9, 54.0, 48.6, 43.3 and 43.1 kg kg^−1^, respectively, and it decreased with increased straw return amount. The AE of different NSR was shown in the following order: NSR_3_ (19.2 kg kg^−1^) > NSR_2_ (19.0 kg kg^−1^) > NSR_4_ (18.9 kg kg^−1^) > NSR_5_ (18.8 kg kg^−1^) > NSR_0_ (18.6 kg kg^−1^) > NSR_1_ (18.4 kg kg^−1^). Furthermore, the AE reached its maximum at a straw return amount of 3–5 t ha^−1^. Similarly, the PFP of different NSR was also shown in the following order: NSR_3_ (42.6 kg kg^−1^) > NSR_2_ (42.4 kg kg^−1^) > NSR_4_ (42.3 kg kg^−1^) > NSR_5_ (42.1 kg kg^−1^) > NSR_0_ (42.0 kg kg^−1^) > NSR_1_ (41.8 kg kg^−1^), and it reached its maximum at a straw return amount of 3–5 t ha^−1^.

**Figure 6:**
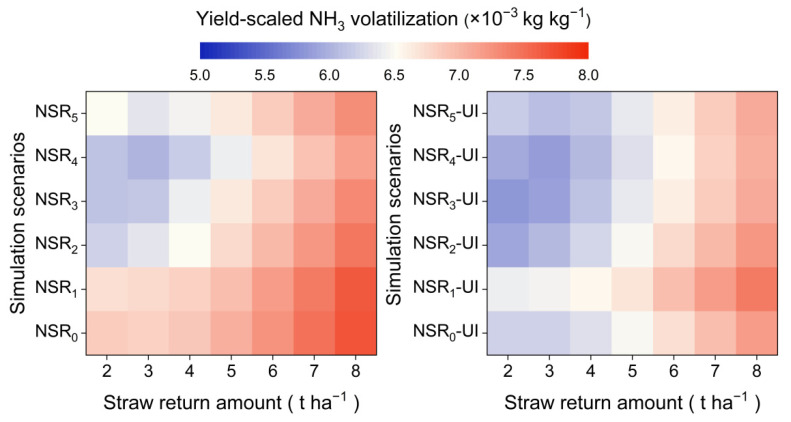
Yield-scaled NH_3_ volatilization under different simulation scenarios. Symbol explanation can be found in Figure 2.

The application of UI and the reduction in straw return amount both contributed to decreasing the yield-scaled NH_3_ volatilization, and NSR_4_ exhibited the lowest yield-scaled NH_3_ volatilization overall (Figure 6). Specifically, the yield-scaled NH_3_ volatilization of NSR_0_, NSR_1_, NSR_2_, NSR_3_, NSR_4_, and NSR_5_ was 7.1, 7.1, 6.8, 6.7, 6.5, and 6.7 × 10^−3^ kg kg^−1^ in the absence of UI application, while that was 6.6, 6.8, 6.5, 6.4, 6.4, and 6.5 × 10^−3^ kg kg^−1^ under the conditions of UI application.

## 3. Discussion

### 3.1. Effects of Straw Returning on Rice Yield and NH_3_ Volatilization

Crop straw is an important agricultural resource, with China producing approximately 855 million tons annually, of which approximately 47% is returned to the field [30]. Straw contains substantial amounts of N, phosphorus, potassium, and other elements. However, due to its slow decomposition, many farmers remain reluctant to return straw to the field, leading not only to resource waste but also exacerbating environmental pollution [30,31]. It is reported that crop straw returning can increase yield by up to 8.64%, thereby contributing to alleviate the growing global demand for food [32]. Straw returning increases the nutrient content and carbon storage in the soil, and enhances water retention capacity, thereby comprehensively improving soil fertility [31,32]. However, straw returning increases the input of exogenous N, enhances the activity of urease, and consequently may lead to increased NH_3_ volatilization [10,33].

In this study, the enhancement effect of straw returning on rice yield was most significant when the straw return amount was less than 4 t ha^−1^. This can be attributed to the fact that straw returning increased N_upt_, which in turn promoted photosynthesis and enhanced the accumulation of dry matter in the grains when the straw return amount was less than 4 t ha^−1^ [9,12]. However, when the straw return amount exceeded 4 t ha^−1^, rice yield increased only slowly or remained stable, while N_upt_ continued to rise. Meanwhile, the PE was decreased with the increased straw return amount. In this study, the maximum yield was below 6.8 t ha^−1^, a relatively lower level, suggesting that rice grains had a lower N requirement [34]. Consequently, the N demand of the grains could be satisfied with a relatively lower straw return amount. Nevertheless, rice straw N uptake might continue to increase with higher straw return amount, resulting in a corresponding increase in overall N_upt_ [34]. Additionally, the straw decomposition process is generally slow and high levels of straw return also may lead to the accumulation of soil-borne diseases, which can negatively impact yield [16,32]. In this study, increased straw return amount primarily enhanced N_upt_ during the TF stage. This might be attributed to the rapid decomposition of easily degradable organic compounds during the initial stage of straw decomposition, and rice had a higher N absorption capacity during the TF stage compared to the BF stage. However, as decomposition progressed, the presence of more resistant compounds such as cellulose and lignin, which were hard to degrade in the subsequent rice growth stages, resulted in limited promotion of N_upt_ during the PF stages [30,35,36].

N loss, through processes such as NH_3_ volatilization, denitrification, N runoff, and N leaching, significantly contributes to low NUE [5]. Straw returning increases the levels of NH_4_^+^-N and NO_3_^−^-N in soil and flooding water, providing more substrates for NH_3_ volatilization and denitrification and raising the risk of N runoff and leaching [10,31,37]. However, straw returning may reduce soil pH and mitigate soil and water loss, potentially reducing N loss [18]. In this study, NH_3_ volatilization increased with higher straw return amount, comprising an average of 61.3% of N loss, which was consistent with previous research (10–80%) [38,39,40]. In paddy fields, high temperatures, prolonged flooding, and the direct application of N fertilizer to flooding water lead to significant NH_3_ volatilization, which becomes a major pathway for N loss [10,41]. Furthermore, straw returning mainly increased NH_3_ volatilization during the BF stage. This could be explained by the fact that, after straw returning, some easily degradable organic matter was rapidly decomposed, which not only enhanced N_upt_ but also increased NH_3_ volatilization [17,35]. Meanwhile, the limited N absorption capacity during the BF stage makes it more prone to substantial NH_3_ volatilization [22]. Overall, excessive straw returning not only had a limited impact on yield increase but also led to greater NH_3_ volatilization. Therefore, the yield-scaled NH_3_ volatilization increased with the increase in straw return amount.

### 3.2. Effects of Urease Inhibitor on Rice Yield and NH_3_ Volatilization

Once applied to the soil, urea undergoes rapid hydrolysis driven by urease, particularly under hot and humid conditions and when applied at the soil surface, which results in substantial NH_3_ volatilization [6,21]. NH_3_ volatilization induces the depletion of available N in soil, consequently constraining rice yield formation. Meanwhile, volatilized NH_3_ can deposit into adjacent water bodies, potentially triggering eutrophication that further threats the sustainable productivity of paddy ecosystems [6,42]. Urease is a hydrolytic enzyme derived from microbial cells and plant and animal residues, and urease activity can be effectively suppressed by UIs, thereby reducing NH_3_ volatilization and improving N uptake and utilization by crops [33,43]. Currently, the main UIs include N-(n-butyl) thiophosphorictriamide (NBPT), phenyl phosphorodiamidate, and hydroquinone. This study employed NBPT, which is considered one of the most effective options for reducing NH_3_ volatilization and increasing crop yield [19].

NBPT prolongs N release from fertilizer, sustaining soil N availability over time and thereby supporting N_upt_ and increasing yield [10,20]. In this study, the impact of the application of UI on N_upt_ and yield was negligible. This effect likely arose from the single NBPT application at the BF stage, during which rice has a relatively lower N uptake capacity compared to the TF and PF stages. Moreover, the efficacy of NBPT in paddy field is sensitive to temperature, and previous studies have shown that high temperatures accelerated NBPT degradation, thereby diminishing its enhancements on N_upt_ and yield [44]. Additionally, urease activity is elevated under straw incorporation conditions, which can also limit the efficacy of NBPT [33].

UIs serve as a key field management practice for mitigating N pollution in farmland. Previous meta-analyses have shown that the application of NBPT can reduce NH_3_ volatilization by 15.2–61% compared to treatments without NBPT [45,46]. In this study, the application of NBPT resulted in an average reduction in NH_3_ volatilization of 2.0–8.5%, indicating a lower efficacy of NBPT. Meanwhile, the increase in straw return amount reduced the NH_3_ volatilization reduction effect of NBPT in this study. On the one hand, NBPT was applied only during the BF stage, resulting in a limited impact on NH_3_ volatilization during the TF and PF stages. In this study, the high-temperature and high-humidity conditions accelerated the degradation of NBPT, thereby diminishing its efficacy [33,44]. The enhancement of microbial growth induced by straw returning, combined with the higher moisture content on the crop residue surface compared to the soil, collectively contributed to a significant increase in urease activity [47]. Additionally, NBPT must convert to N-butyl-phosphorothioic triamide oxide, an active form responsible for urease inhibition, to effectively suppress urease activity. However, this conversion process is hindered under the typical anaerobic conditions of paddy fields, which further limits NBPT’s capacity to reduce NH_3_ volatilization in this study [48].

### 3.3. Effects of N Split Application Ratio on Rice Yield and NH_3_ Volatilization

Inappropriate N fertilizer management practices significantly contribute to low NUE and environmental issues [5,7]. Aligning N supply with crop demand is essential to enhance both crop yield and NUE [22]. In China, the NSR is frequently not tailored to align with the real-time nutrient demands of crops [23]. It is reported that in China’s intensive rice-cropping regions, reducing N fertilizer application during early growth stage could sustain rice yield while considerably enhancing NUE [8,22,23].

In this study, increasing the PF proportion was most beneficial for enhancing N_upt_, thereby increasing yield. During the early growth stage, rice roots are relatively undeveloped and show limited N absorption capacity [22,36]. As root development progresses, the N absorption capacity of the root system correspondingly increases [23,36]. Hence, high N fertilizer rate during early growth stage result in a misalignment between crop demand and N supply [49]. Increasing N fertilizer rate during late growth stage can enhancing rice grain weight and filling rate, which directly promotes yield increase [12,22]. Meanwhile, increasing N availability in late growth stage enhances photosynthetic activity and delays leaf senescence, thus supporting crop productivity during the late growth stage [22]. However, the yield of NSR_4_ and NSR_5_ was lower than that of NSR_3_, suggesting that excessively reducing the proportions of BF and TF may result in insufficient nutrient supply during the vegetative growth stage, ultimately affecting yield formation in late growth stages. Furthermore, the increase in straw return amount weakened the effect of NSR on N_upt_ during the BF and PF stages. This can be explained by the fact that under high straw return amount, soil N supply remains relatively sufficient across different NSR, thereby weakening the impact of NSR on N_upt_ [31,37].

Optimizing the NSR not only aligns with N demand for rice across different growth stages, but also contributes to reduce N loss. In this study, increasing the PF proportion reduced NH_3_ volatilization during the BF and TF stages, ultimately leading to a decrease in total NH_3_ volatilization over the entire growth period. During the early growth stage, rice N uptake capacity is limited, resulting in a greater NH_3_ volatilization proportion of N fertilizer rate compared to in the later stage [10,36]. Meanwhile, the effect of NSR in NH_3_ volatilization reduction decreased with the increased straw return amount. NSR not only directly affect the supply of NH_4_^+^-N but also influence urease activity. At lower straw return amount, urease activity is generally low, and changes in NSR will significantly affect urease activity. However, at higher straw return amount, urease activity remains high across different NSR, which weakens the effect of NSR in NH_3_ volatilization reduction [33,41]. Overall, NSR_3_ combined with a straw return amount of 3 t ha^−1^ under the conditions of UI application was the optimal field management practice, which not only achieved the highest yield but also maintained low NH_3_ volatilization and high NUE.

### 3.4. Limitations of the Model Simulation

In this study, the model employs the NH_3_ volatilization calculation method established by Freney et al. [50], which specifically incorporates paddy field characteristics by accounting for mineral N concentration, water temperature, and pH in the ponding water layer. However, several critical meteorological factors including air temperature, wind speed and humidity are not included in the current model, which influence the diffusion rate of NH_3_ from water to air [6]. The pH of ponding water undergoes dynamic changes during urea hydrolysis and NH_3_ volatilization, which in turn affects NH_3_ volatilization, but the present model does not take into account the dynamic process of pH during these processes. Although most volatilized NH_3_ in paddy field directly enters the atmosphere, rice leaves can absorb a portion through stomata, with increased uptake occurring under higher NH_3_ concentrations [51]. Furthermore, the photorespiration process in plants generates NH_3_ that may escape through leaf stomata if not efficiently reassimilated in chloroplasts. During leaf senescence, protein degradation produces amino acids and amine compounds, a portion of which further hydrolyze into NH_3_ and volatilizes into the atmosphere [52]. These mechanisms demonstrate that rice plants exhibit bidirectional NH_3_ exchange-both absorbing and emitting NH_3_. However, the current model does not consider these effects of leaves on NH_3_ volatilization. In the future, we will strive to address the limitations of the model in these aspects to more reliably evaluate the impacts of field management practices on NH_3_ volatilization in paddy fields.

## 4. Materials and Methods

### 4.1. The Study Site

The field experiment was conducted at the Agricultural Resource Conservation and Utilization Experimental Station of the Sichuan Academy of Agricultural Sciences, located in Ziyang, Sichuan Province (104°32′–104°35′ E; 30°05′–30°07′ N). This site is situated in the central area of the purple soil hilly region of Sichuan Basin at an elevation of approximately 400 m, within the subtropical monsoon continental climate zone. The study site has an average temperature of 17 °C and receives approximately 1250 h of sunlight annually. The study site receives approximately 950 mm of rainfall annually, with the majority occurring in the summer and autumn seasons. The predominant soil type is purple soil, developed from the Jurassic Suining Formation parent material. Rice–rapeseed rotation is one of the main cultivation practices in the area. Soil samples from the 0–20 cm depth were collected before the experiment to determine the basic soil properties. The soil had a pH of 7.73, with soil organic matter content 32.83 g kg^−1^, total N content 2.16 g kg^−1^, total phosphorus content 1.10 g kg^−1^, and total potassium content 21.91 g kg^−1^.

### 4.2. Experimental Design and Field Management

The field experiment was conducted from May 2018 to September 2019. The experiment comprised eight treatments: control (CK), application of urea only (U), application of urea combined with rapeseed straw returning at amounts of 2, 5, and 8 t ha^−1^ (US_2_, US_5_, and US_8_), and urea mixed with a UI along with rapeseed straw returning at the same amounts (UIS_2_, UIS_5_, and UIS_8_). Each treatment was replicated three times, with each plot measuring 30 m^2^. The UI used is N-(n-butyl) thiophosphorictriamide (NBPT), applied only with the BF at a ratio of 1%.

In 2018 and 2019, rice was transplanted on 30 May and 2 June, respectively, with harvesting conducted on 16 September and 20 September. Urea was applied at a rate of 150 kg N ha^−1^, with an NSR for BF, tiller fertilizer (TF), and panicle fertilizer (PF) of 2:1:2. P_2_O_5_ and K_2_O were applied only for BF at rates of 75 kg ha^−1^ and 105 kg ha^−1^, respectively. All treatments maintained identical irrigation conditions, with the flooding water depth in the paddy field kept at 0–5 cm from transplanting until two weeks before harvest. The evapotranspiration amount during the 2018 and 2019 rice growing seasons was approximately 350 mm and 310 mm, respectively. Rapeseed straw was chopped into 5 cm segments and incorporated into the 0–20 cm soil layer before rice transplanting. The carbon and N contents of rapeseed straw are 40.6% and 0.42%, respectively. Specific agricultural operations were implemented as described by Wang et al. [10].

### 4.3. Observations and Measurement Methods

The NH_3_ volatilization was measured using the dynamic chamber method, employing a dynamic plexiglass cylindrical chamber with a height of 18 cm and an inner diameter of 25 cm. Daily measurements were taken between 8:00 and 10:00 am and 3:00 and 5:00 pm. Samples were collected daily until the different treatments were consistent with the CK after each fertilization. Samples were absorbed using a boric acid solution with a concentration of 2%. The NH_4_^+^-N concentration was quantified using 0.01 mol L^−1^ HCL, with a bromocresol green–methyl red mixture as the indicator [53,54].

At the harvest stage, rice grain and straw samples were collected and fixed at 105 °C for 30 min, followed by drying at 75 °C until constant weight was achieved, to determine yield and ADM. Ten random rice samples were selected from each plot to measure the N_upt_ using a flow analyzer (AA3, Bran+Luebbe Inc., Norderstedt, Germany) after digestion with H_2_SO_4_-H_2_O_2_ [55]. The daily meteorological data, including temperature, relative humidity, wind speed, sunshine hours, and rainfall, were recorded at a nearby meteorological station.

Furthermore, the N physiological efficiency (PE, kg kg^−1^), agronomic efficiency (AE, kg kg^−1^), partial factor productivity (PFP, kg kg^−1^), and yield-scaled NH_3_ volatilization (×10^−3^ kg kg^−1^) served as indicators for assessing NUE [56].

(1) $$\mathrm{PE}=\frac{{\mathrm{Yield}}_{N}-{\mathrm{Yield}}_{\mathrm{CK}}}{N_{\mathrm{upt}-N}-N_{\mathrm{upt}-\mathrm{CK}}}$$

(2) $$\mathrm{AE}=\frac{{\mathrm{Yield}}_{N}-{\mathrm{Yield}}_{\mathrm{CK}}}{N\mathrm{application}\;\mathrm{rate}}$$

(3) $$\mathrm{PFP}=\frac{{\mathrm{Yield}}_{N}}{N\mathrm{application}\;\mathrm{rate}}$$

(4) $$\mathrm{Yield}-\mathrm{scaled}\;{\mathrm{NH}}_{3}\;\mathrm{volatilization}=\frac{{\mathrm{NH}}_{3}\;\mathrm{volatilization}\times 10^{3}}{\mathrm{Yield}}$$

where N_upt-N_ and N_upt-CK_ represent the N uptake by the crop in the N-fertilized treatment and CK, respectively; Yield_N_ and Yield_CK_ denote the rice yield for the N-fertilized treatment and CK, respectively; N application rate represents the total N from both Urea and rapeseed straw.

### 4.4. WHCNS Model

The WHCNS model runs on a daily time step and encompasses processes such as soil water movement, crop growth and N uptake, N mineralization, urea hydrolysis, NH_3_ volatilization, nitrification, denitrification, and organic matter turnover. The model estimates crop evapotranspiration using the Penman–Monteith equation [57]. Under unsaturated conditions, the model simulates soil water infiltration and redistribution using the Green–Ampt model and Richards’ equation, while Darcy’s law is applied under saturated conditions [58]. The crop growth processes such as the accumulation of photosynthetic product, yield formation, and leaf growth were simulated by the improved PS123 model [59]. The simulation of soil C and N cycling process are based on the Daisy model [60].

In this model, the hydrolysis of urea typically occurs within a few days in hot seasons. The hydrolysis process of urea applied with UI can be described by the following equation:

(5) $$S_{hys}=N_{urea}\times \left[1-\exp\left(-5.0\times WFPS\times K_{h}\right)\right]$$

where *S_hys_* is the urea hydrolysis rate (μg cm^−3^ d^−1^), *N_urea_* is the urea content in the soil (μg cm^−3^), *WFPS* is the water-filled pore space (%), which is calculated by dividing the soil volumetric water content by the soil porosity, and *K_h_* is the urea hydrolysis coefficient (d^−1^). In this study, we adjusted the value of *K_h_* to modify the hydrolysis rate of urea for the treatments involving the UI. More detailed information regarding other processes in the model is available in Liang et al. [28] and Shi et al. [6].

The calculation of daily NH_3_ volatilization (*N_v_*) in paddy field can be described by the following equation [50]:

(6) $$N_{v}=\frac{K_{v}\times C_{p}\times f(T)}{1+10^{\left(0.09018+\frac{2729.92}{T+273.15}\times pH\right)}}$$

(7) f(T)=0.25×exp(0.0693×T)

where *K_v_* is NH_3_ volatilization first-order kinetic constant (d^−1^), *C_p_* is the mineral N content of surface ponding water (mg N L^−1^), *T* is the temperature of surface ponding water (°C), *pH* is the pH value of surface ponding water, and *f*(*T*) is the temperature function.

### 4.5. Model Calibration, Validation, and Evaluation

Based on the calibration results of the WHCNS model parameters from adjacent paddy fields [61], this study used a trial-and-error method to calibrate the crop parameters, soil hydraulic parameters, and N transformation parameters by the measured rice yield, ADM, N_upt_, and NH_3_ volatilization from 2018. On this basis, the urea hydrolysis coefficient with UI application was calibrated using the measured data from treatments involving the UI application in 2018. Then, the model was validated using data from 2019. The *K_h_* was set to 0.007 d^−1^ under the application of 1% UI, and other calibration results of the parameters are presented in Table 2.

The normalized root mean squared error (*nRMSE*), index of agreement (*IA*), and Nash–Sutcliffe efficiency (*NSE*) were used as indices for evaluating model performance:

(8) $$nRMSE=\frac{\sqrt{\frac{1}{n}\times \sum_{i=1}^{n}{\left(S_{i}-O_{i}\right)}^{2}}}{O}\times 100$$

(9) $$IA=1-\frac{\sum_{i=1}^{n}{\left(S_{i}-O_{i}\right)}^{2}}{\sum_{i=1}^{n}{\left(\left|S_{i}-O\right|+\left|O_{i}-O\right|\right)}^{2}}$$

(10) $$NSE=1-\frac{\sum_{i=1}^{n}{\left(S_{i}-O_{i}\right)}^{2}}{\sum_{i=1}^{n}{\left(O_{i}-O\right)}^{2}}$$

where *n* is the number of values; *S_i_* is the simulated values; *O_i_* is the observed values; and *O* is the mean of the observed values. An *nRMSE* of less than 15% indicates good simulation performance, while a value between 15% and 30% represents moderate performance, and a value greater than 30% suggests poor simulation performance. *IA* ≥ 0.75 and *NSE* ≥ 0 are the minimum thresholds for crop growth simulation. *IA* ≥ 0.60 and *NSE* ≥ −1 are the minimum thresholds for N output simulation [62].

### 4.6. Simulation Scenarios

The following simulation scenarios were established in 2019 after calibrating and validating the WHCNS model: (1) Straw return amounts were established at seven levels: 2, 3, 4, 5, 6, 7, and 8 t ha^−1^. (2) Adjusting NSR to 1:2:2 (NSR_1_), 2:3:5 (NSR_2_), 1:1:3 (NSR_3_), 1:2:7 (NSR_4_), and 2:1:7 (NSR_5_) on the basis of 2:1:2 (NSR_0_). A total of 84 simulation scenarios were designed: 7 straw return amounts × 6 NSR × 2 urea application methods (with or without UI application).

## 5. Conclusions

This study employed the WHCNS model to simulate the effects of straw returning, UI, and NSR on rice yield, N_upt_, and NH_3_ volatilization based on field experiment in the Sichuan Basin. Excessive straw return amount not only showed limited benefits for increasing rice yield, but also exacerbated NH_3_ volatilization. Increasing the PF proportion enhanced N_upt_ during the PF stage, thereby promoting yield improvement, while the effect of UI on N_upt_ and yield enhancement was minimal. Increasing the PF proportion and the application of UI reduced NH_3_ volatilization during the BF or TF stage, thereby reducing NH_3_ volatilization over the entire growth period. Meanwhile, the NH_3_ volatilization reduction effects of both practices diminished with the increased straw return amount. Additionally, NSR_3_ achieved the highest yield while maintaining a high level of NUE. The combination of NSR_3_ and UI, with a straw return amount of 3 t ha^−1^, was identified as the optimal field management practice in purple soil area, ensuring both food security and environmental benefits.

## Figures and Tables

**Table 1:** The simulation evaluation indicators for the WHCNS model.

Indicators	2018			2019		
	*nRMSE* (%)	*IA* (−)	*NSE* (−)	*nRMSE* (%)	*IA* (−)	*NSE* (−)
Rice yield	7.3	0.96	0.74	6.3	0.94	0.60
ADM	7.2	0.93	0.57	1.9	0.99	0.96
N_upt_	10.0	0.93	0.57	12.8	0.87	0.29
NH_3_ volatilization	7.2	0.99	0.94	15.1	0.95	0.72

ADM: aboveground dry matter; N_upt_: crop N uptake.

**Table 2:** The calibration results of WHCNS model parameters.

Parameters		Value
Soil hydraulic parameters	Saturated hydraulic conductivity: *K_s_*_1_, *K_s_*_2_, and *K_s_*_3_ (cm d^−1^)	1, 0.6, 0.7
	Saturated water content: *θ_s_*_1_, *θ_s_*_2_, and *θ*_s3_ (cm^3^ cm^−3^)	0.65, 0.50, 0.43
	Field capacity: *FC*_1_, *FC*_2_, and *FC*_3_ (cm^3^ cm^−3^)	0.35, 0.25, 0.24
	Wilting point water content: *WP*_1_, *WP*_2_, and *WP*_3_ (cm^3^ cm^−3^)	0.17, 0.15, 0.13
Crop parameters	Base temperature: *T_b_* (°C)	10
	Accumulated temperature: *T_s_* (°C)	1630
	Extinction coefficient: *K_e_* (−)	0.5
	Crop coefficient in the initial stage: *K_ini_* (−)	0.8
	Crop coefficient in the middle stage: *K_mid_* (−)	1.4
	Crop coefficient in the end stage: *K_end_* (−)	0.7
	Maximum specific leaf area: *SLA_max_* (m^2^ kg^−1^)	22
	Minimum specific leaf area: *SLA_min_* (m^2^ kg^−1^)	10
	Maximum root depth: *R_max_* (m)	0.5
	Crop maximum critical N concentration: *N_crit_* (%)	2.5
N transformation parameters	Maximum nitrification rate: *V_n_* (mg L^−1^ d^−1^)	70
	Nitrification half saturation constant: *K_n_* (mg L^−1^)	70
	Denitrification empirical proportionality factor: *K_d_* (−)	0.5
	Denitrification empirical constant: *A_d_* (mg mg^−1^)	0.02
	Ammonia volatilization first-order kinetic constant: *K_v_* (d^−1^)	0.095
	Hydrolysis coefficient of urea: *K_h_* (d^−1^)	0.05
	Hydrolysis coefficient of urea in treatment with 1% urease inhibitors: *K_h_* (d^−1^)	0.007

_1_, _2_, and _3_ represent soil depths of 0–20 cm, 20–40 cm, and 40–80 cm, respectively.

## Data Availability

Data are contained within this article.

## Supplementary Materials

The following supporting information can be downloaded at: https://www.mdpi.com/article/10.3390/plants14121744/s1, Figure S1: The measured and simulated values of rice yield; Figure S2: The measured and simulated values of rice ADM; Figure S3: The measured and simulated values of crop N uptake; Figure S4: The measured and simulated values of NH_3_ volatilization dynamics in 2018; Figure S5: The measured and simulated values of NH_3_ volatilization dynamics in 2019; Figure S6: The measured and simulated values of cumulative NH_3_ volatilization.
