# Supplementary material for: Modeling the Combined Effects of Straw Returning, Urease Inhibitors, and Nitrogen Split Application on Rice Yield and Ammonia Volatilization in Purple Soil Area
Source: Plants (Basel). 2025 Jun 6;14(12):1744. doi: 10.3390/plants14121744 (PMC12196444; doi:10.3390/plants14121744)
Supplement: Supplementary file 1 [file plants-14-01744-s001.zip › plants-3623681-supplementary.pdf]

Modeling the Combined Effects of Straw Returning, Urease Inhibitors, and Nitrogen Split Application on Rice Yield and Ammonia Volatilization in Purple Soil Area

Tianxiang Xu [1], Hong Wang [2], Huirong Hao [1], Chaowen Lin [2] and Kelin Hu [1,*]

[1] College of Land Science and Technology, China Agricultural University, Key Laboratory of Arable Land Conservation (North China), Ministry of Agriculture and Rural Affairs, Beijing 100193, China
[2] Institute of Agricultural Resources and Environment, Sichuan Academy of Agricultural Sciences, Chengdu 610066, China
* Correspondence: hukel@cau.edu.cn

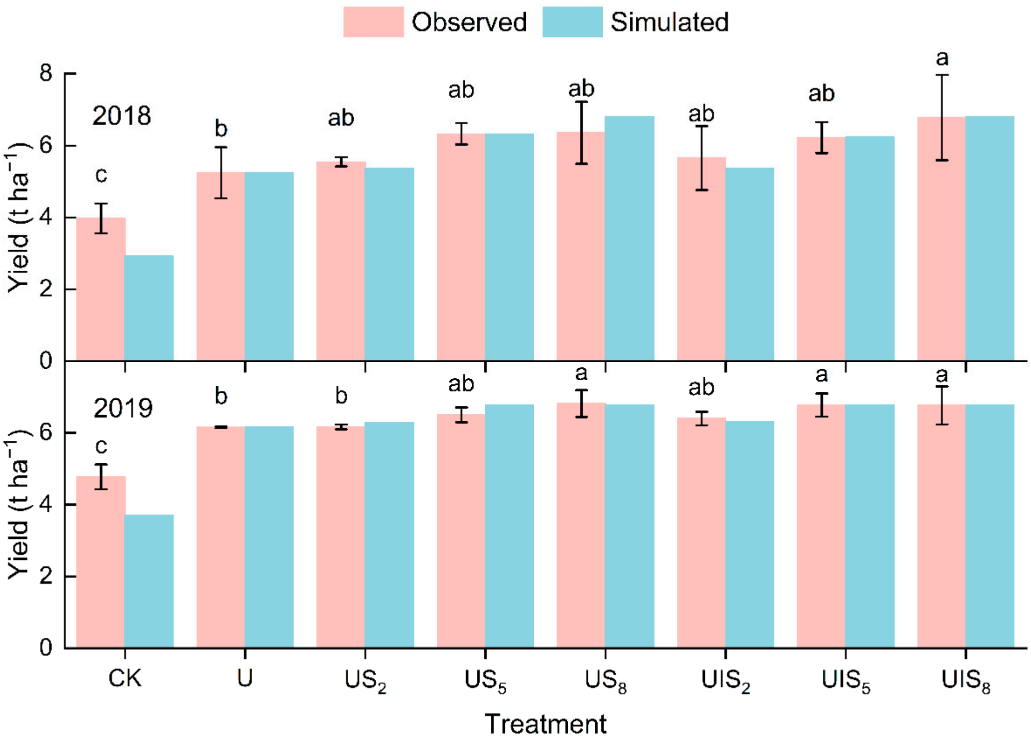

Figure S1 The measured and simulated values of rice yield. CK: control; U: urea; US_2, US_5, and US_8: urea combined with rapeseed straw returning at amounts of 2, 5, and 8 t ha^{-1}, respectively; UIS_2, UIS_5, and UIS_8: urea incorporated with a urease inhibitor at the same straw return amounts. Identical letters within the same year indicate no significant differences in rice yield.

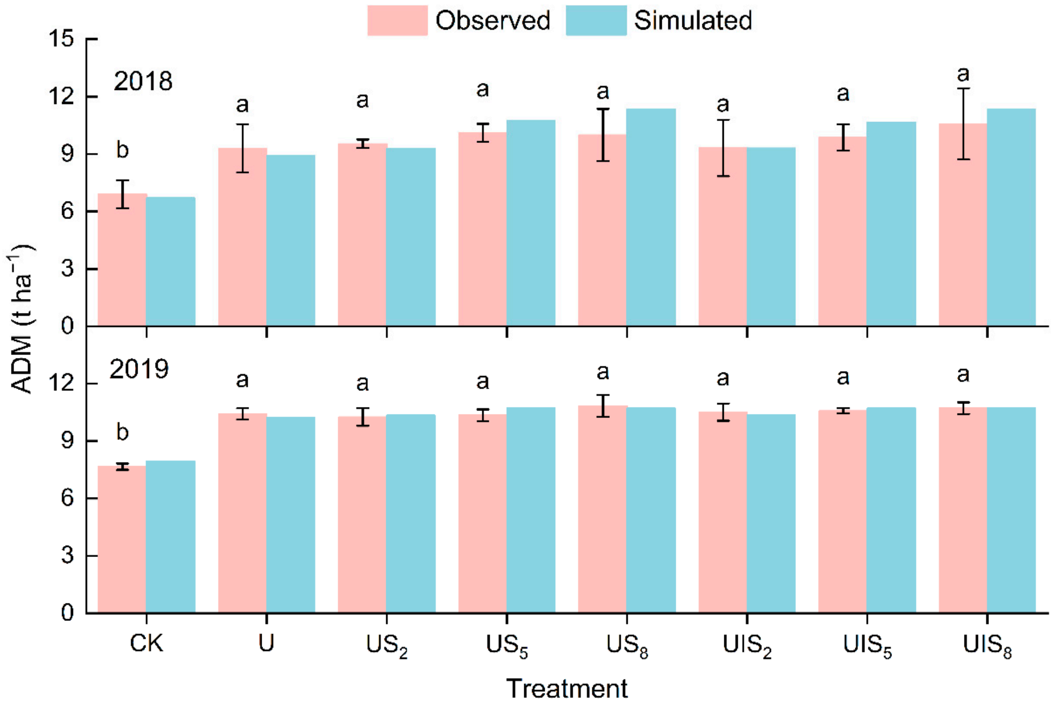

Figure S2 The measured and simulated values of rice aboveground dry matter (ADM). The symbol explanation can be referred to Figure S1. Identical letters within the same year indicate no significant differences in ADM.

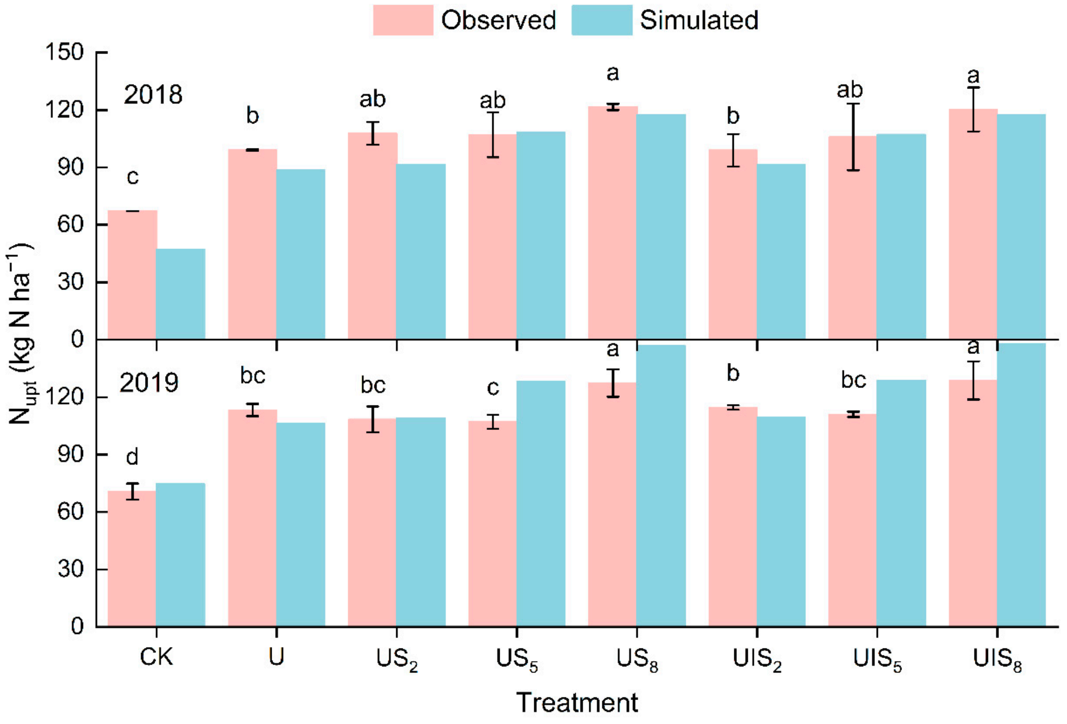

Figure S3 The measured and simulated values of crop N uptake (N_{upt}). The symbol explanation can be referred to Figure S1. Identical letters within the same year indicate no

significant differences in N_{upt}.

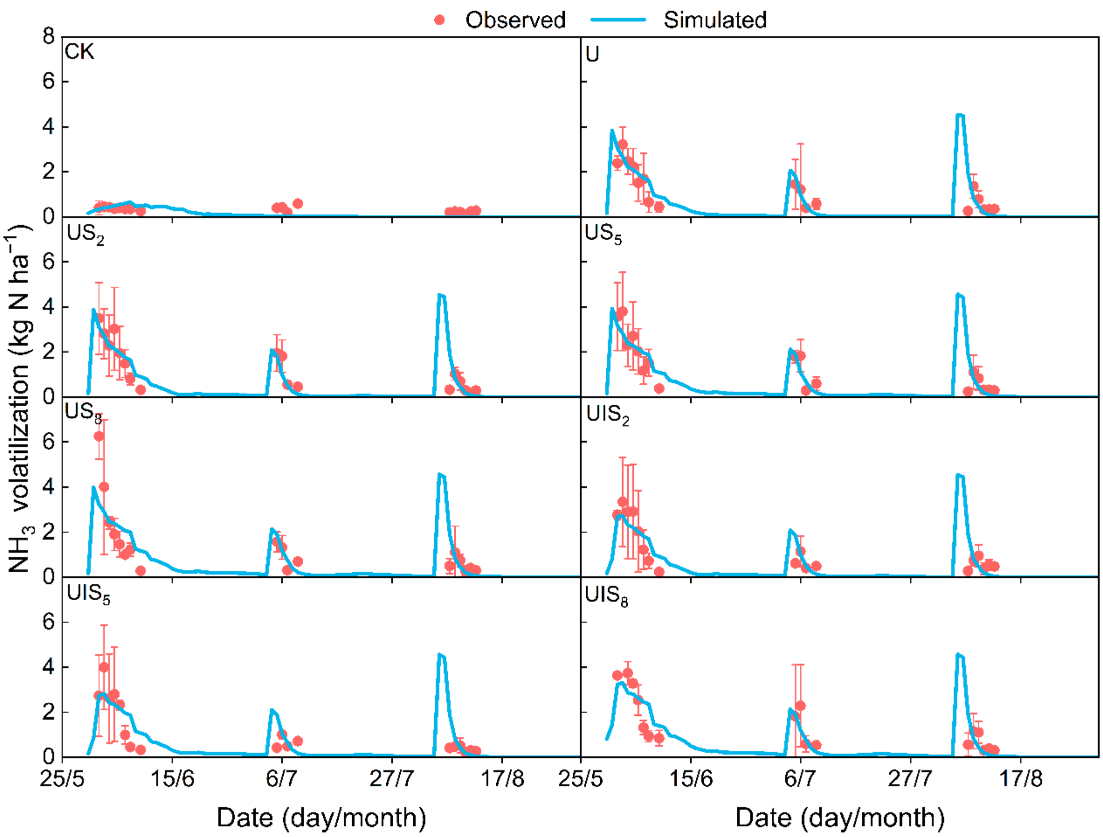

Figure S4 The measured and simulated values of NH_3 volatilization dynamics in 2018. The symbol explanation can be referred to Figure S1.

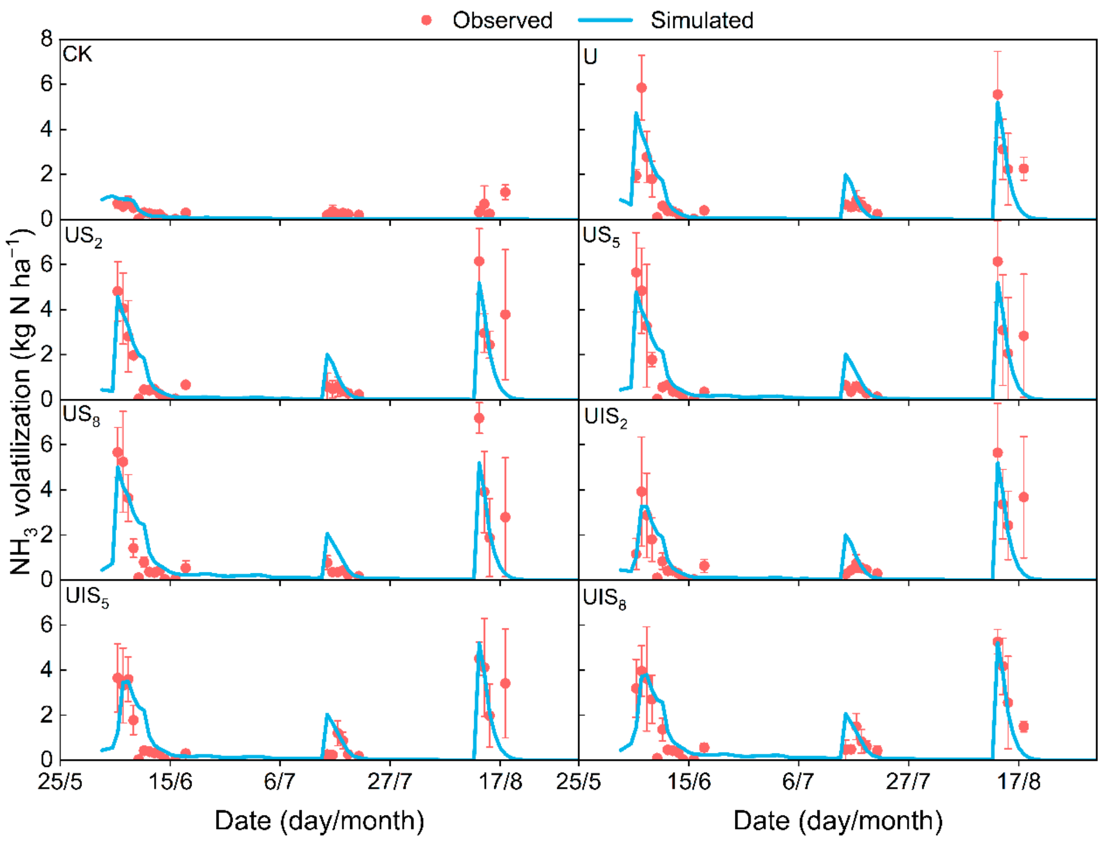

Figure S5 The measured and simulated values of NH_3 volatilization dynamics in 2019. The symbol explanation can be referred to Figure S1.

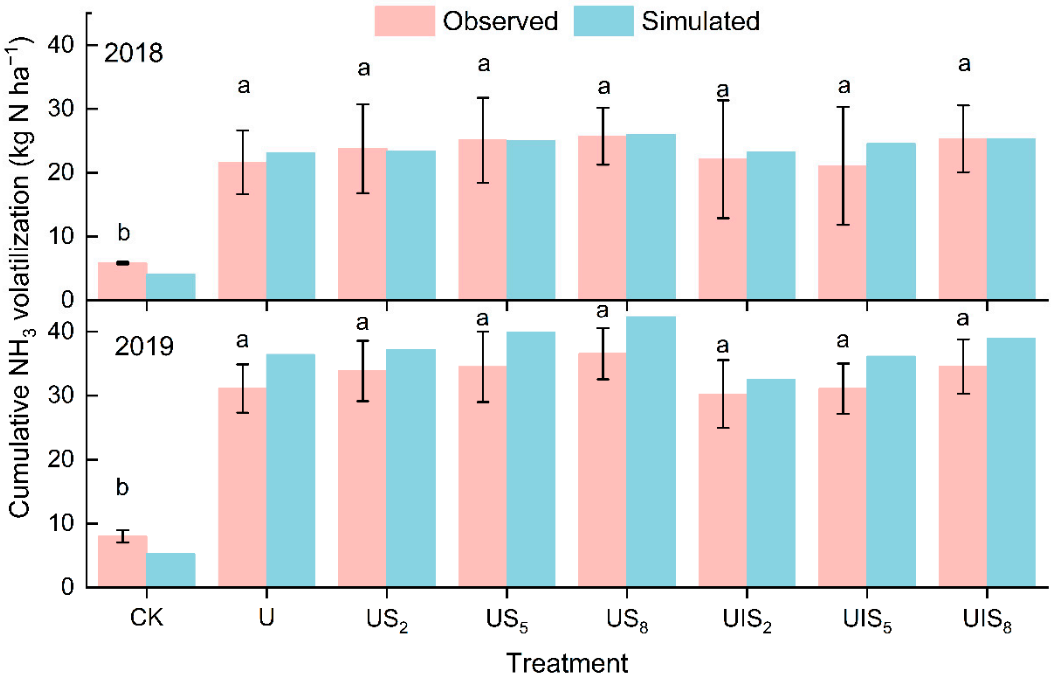

Figure S6 The measured and simulated values of cumulative NH_3 volatilization. The symbol explanation can be referred to Figure S1. Identical letters within the same year indicate no

significant differences in cumulative NH_3 volatilization.
